# The Role of *torS* and *torT* Transcription in Driving a Bet‐Hedging Strategy for Trimethylamine *N*‐Oxide Reductase Expression in *Escherichia coli*

**DOI:** 10.1111/mmi.70103

**Published:** 2026-08-01

**Authors:** Erin L. Mettert, Jeffrey N. Carey, Wilma Ross, Mark Goulian, Patricia J. Kiley

**Affiliations:** ^1^ Department of Biomolecular Chemistry, School of Medicine and Public Health University of Wisconsin‐Madison Madison Wisconsin USA; ^2^ Graduate Group in Biochemistry and Molecular Biophysics, Perelman School of Medicine University of Pennsylvania Philadelphia Pennsylvania USA; ^3^ School of Veterinary Medicine University of Pennsylvania Philadelphia Pennsylvania USA; ^4^ Department of Bacteriology, College of Agriculture and Life Sciences University of Wisconsin‐Madison Madison Wisconsin USA; ^5^ Department of Biology University of Pennsylvania Philadelphia Pennsylvania USA; ^6^ Department of Physics and Astronomy University of Pennsylvania Philadelphia Pennsylvania USA

**Keywords:** bet‐hedging, divergent promoters, IscR, regulation, TMAO reduction, transcription

## Abstract

In *Escherichia coli* , cell‐to‐cell variability in aerobic expression of trimethylamine *N*‐oxide (TMAO) reductase serves as a bet‐hedging strategy, enabling a subset of cells to continue growing upon a shift to anaerobic conditions in the presence of TMAO. This variability has been attributed to the low abundance of TorS and TorT signaling proteins, which along with TorR, regulate transcription of *torCAD* encoding TMAO reductase. Here, we characterize the divergent promoters that drive expression of the bipartite sensor and kinase, TorT and TorS. In vitro transcription analysis identified two transcription start sites for *torS* and one start site for *torT*, all located in close proximity. Notably, the RNA polymerase‐σ^70^–10 element of the upstream *torS* promoter overlaps that of the *torT* promoter, defining it as a bidirectional promoter. We further demonstrate that these two promoters are subject to repression by the global regulator IscR, which binds to a site that partially overlaps the shared −10 elements. IscR binding antagonizes RNAP‐σ^70^ binding, limiting transcription of both promoters and contributing to low *torS/torT* expression. Finally, we show that a single base substitution within the overlapping −10 elements increases *torS/torT* transcription by at least 4‐fold and abolishes cell‐to‐cell variability in *torCAD* expression, despite this promoter variant remaining sensitive to IscR‐mediated repression. Together, these findings indicate that promoter strength and transcriptional repression are critical components for regulated bet‐hedging, enabling *E. coli* to optimize energy metabolism in response to fluctuations in oxygen availability and TMAO levels.

## Graphical abstract

Low basal activity of divergent promoters driving transcription of the trimethylamine N‐oxide (TMAO) sensor TorT and kinase TorS is integral to an O_2_‐regulated bet‐hedging strategy for continued *E. coli* growth upon the onset of anaerobiosis. This, along with repression by IscR, promotes noise in the TorS to TorT ratio, which is critical for adaptation to anaerobic TMAO metabolism. Strengthening these promoters eliminates noise in the TorS to TorT ratio and consequently bet‐hedging.

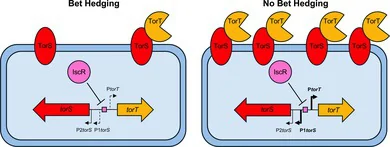

## 1 Introduction

To cope with abrupt environmental fluctuations, bacteria have evolved a range of regulatory strategies that enable rapid phenotypic adjustments. One such strategy, bet‐hedging, generates phenotypic heterogeneity within a population ensuring that a subset of cells is preadapted for sudden environmental shifts. Although this heterogeneity can result in some cells exhibiting phenotypes that reduce fitness under existing conditions, these same phenotypes can confer a substantial advantage in alternative environments, thereby increasing the likelihood that at least part of the population will thrive when conditions change rapidly (Veening et al. 2008; Thattai and Van Oudenaarden 2004; Kussell and Leibler 2005). Here, we build on our prior work to further characterize a bet‐hedging mechanism in *Escherichia coli* , in which variable expression of the trimethylamine *N*‐oxide (TMAO) respiratory system during aerobic growth enables a subset of cells to survive an abrupt loss of O_2_ (Carey et al. 2018).

Although aerobic respiration conserves more energy and is generally preferred, *E. coli* can switch to fermentation or alternate respiratory pathways, such as TMAO reduction, when O_2_ becomes limiting. TMAO, derived from dietary metabolism, is reduced to trimethylamine by TMAO reductase, encoded by the *torCAD* operon (Mccrindle et al. 2005; Cheng and Weiner 2007). In contrast to other alternate respiratory pathways that are selectively expressed anaerobically, mean *torCAD* expression in the presence of TMAO was shown to be O_2_‐independent (Unden and Bongaerts 1997; Ansaldi et al. 2007). Nevertheless, while *torCAD* expression is relatively uniform across a cellular population under anaerobic conditions, it is highly variable from cell to cell during aerobic growth (Roggiani and Goulian 2015; Carey et al. 2018). Importantly, only the aerobic subpopulation with a history of elevated *torCAD* expression continues growing after an abrupt shift to anaerobiosis in the presence of TMAO (Carey et al. 2018).

Expression of *torCAD* is regulated at the transcriptional level by the TorT‐TorS‐TorR signal transduction system (Figure 1A). Upon binding periplasmic TMAO, TorT induces autophosphorylation of membrane‐bound TorS, which subsequently transfers the phosphate to TorR, enabling this cytoplasmic transcription factor to activate *torCAD* transcription (Baraquet et al. 2006; Jourlin, Bengrine, et al. 1996; Jourlin, Simon, et al. 1996; Jourlin et al. 1997; Simon et al. 1994, 1995; Ansaldi et al. 2000). However, in the absence of TMAO, the basal phosphatase activity of TorS instead dephosphorylates TorR‐P, keeping *torCAD* expression low (Ansaldi et al. 2001). The dual functionality of TorS further suggests that *torCAD* transcription is sensitive to the cellular TorS/TorT protein ratio even in the presence of TMAO as this balance likely determines whether TorS acts predominantly as a kinase or a phosphatase for TorR.

**FIGURE 1:**
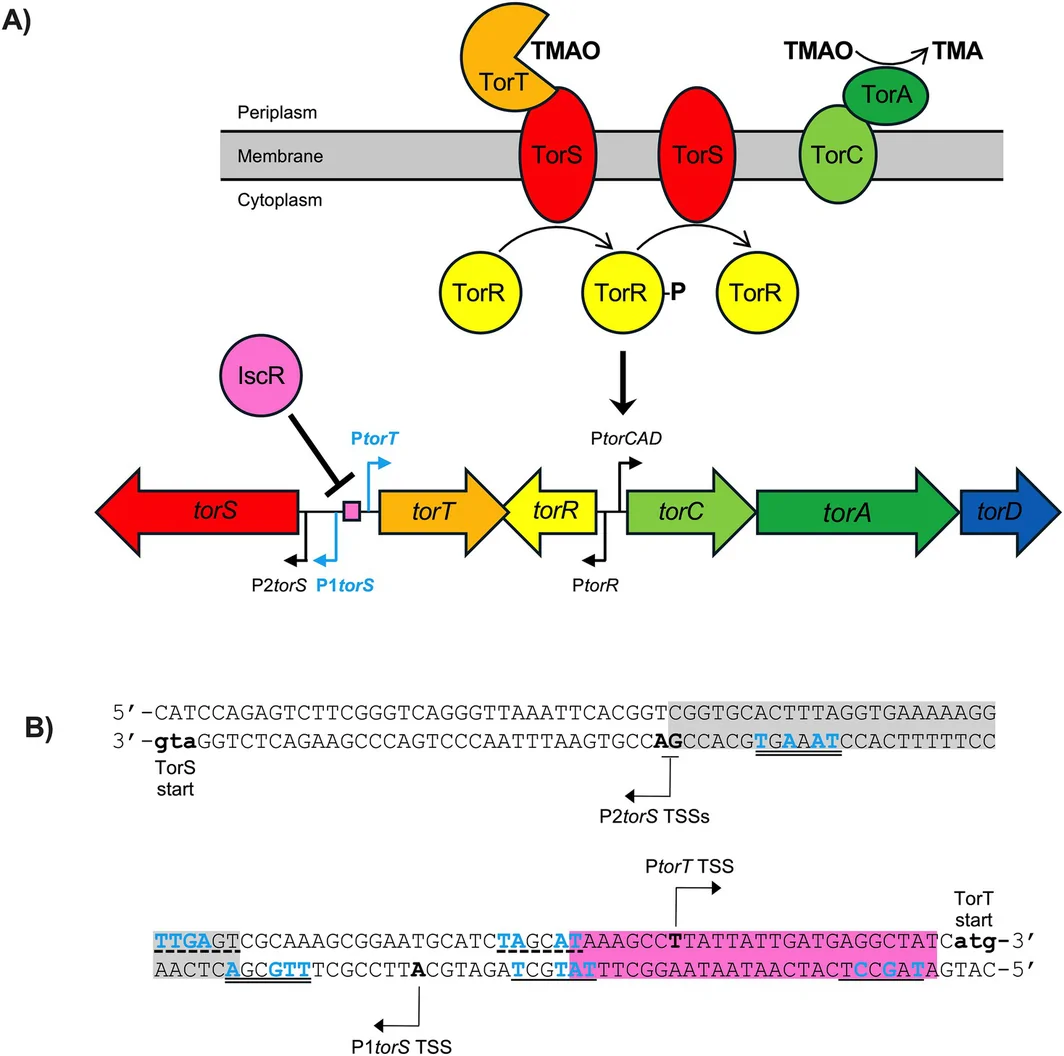
(A) Model for *torCAD* transcriptional regulation by the TorS/TorT ratio. When bound to TMAO, TorT promotes autophosphorylation of TorS. TorS transfers its phosphoryl group to TorR, and TorR‐P activates transcription of *torCAD*, encoding TMAO reductase. In the absence of TMAO, TorS dephosphorylates TorR, and *torCAD* transcription remains low. Under aerobic conditions IscR binds its recognition motif (pink box) within the intergenic region between *torS* and *torT* to repress transcription from promoters P1*torS* and P*torT* (marked by blue arrows). (B) Shown is the sequence of the *torS*/*torT* intergenic region, encompassing −95 to +23 bp relative to the P*torT* TSS (determined in this study); this sequence corresponds to −40 to +78 bp and −82 to +36 bp relative to the P1*torS* and P2*torS* TSSs, respectively. Start codons are in lower case, TSSs are in bold type, direction of transcription is indicated by arrows, the IscR binding motif is highlighted in pink, and the bacteriophage HK022 *attB* integration site is highlighted in gray. The predicted −10 and −35 RNAP‐σ^70^ recognition elements are underlined for P1*torS* (single), P2*torS* (double), and P*torT* (dotted). Bases matching the consensus −10 and −35 elements (5′TATAAT3′ and 5′TTGACA3′, respectively) are in blue bold type. It should be noted that for P2*torS*, we refer to the A TSS as +1 despite transcription occurring from both the A and G TSSs in vitro.

Notably, TorS and TorT are predicted by ribosome profiling experiments to be present at extremely low levels of four molecules per cell under aerobic conditions (Li et al. 2014). Such low abundance is predicted to generate stochastic fluctuations in the TorS/TorT ratio, leading to variability in TorR‐P levels in the presence of TMAO, and consequently, heterogeneous *torCAD* transcription across a cellular population during aerobic growth. Consistent with this model, increased expression of TorS and TorT from multicopy plasmids appeared to reduce noise in the TorS/TorT ratio, thereby significantly decreasing the cell‐to‐cell variability of *torCAD* transcription (Roggiani and Goulian 2015). Together, these findings suggest that maintaining low cellular concentrations of TorS and TorT promotes transcriptional heterogeneity, potentially serving as a bet hedging strategy.

Indeed, *torS* and *torT* are subject to repression under aerobic growth conditions by the global regulator IscR (Carey et al. 2018). An IscR binding motif was identified in the intergenic region between *torS* and *torT*, which are divergently transcribed (Figure 1B). Importantly, loss of IscR or disruption of its binding site reduces cell‐to‐cell variability in *torCAD* expression, indicating that IscR mediated repression plays a role in this bet‐hedging strategy (Carey et al. 2018). However, the molecular basis of this repression has remained unclear, in part because the promoter architecture governing *torS*/*torT* transcription had not been defined. Previous in vitro studies identified two closely spaced transcription start sites upstream of *torS* (Carey et al. 2019), whereas the start site of the divergently transcribed *torT* was identified by high‐throughput genomic methods (Ju et al. 2019). Notably, sequence comparisons of the *torS*/*torT* intergenic region revealed a high degree of conservation across the *Enterobacteriaceae* (Carey et al. 2018), suggesting that specific promoter features are evolutionarily maintained to keep *torS* and *torT* expression sufficiently low to permit noise in the TorS/TorT ratio and thus enable bet‐hedging.

In this study we defined the promoter architecture controlling *torS* and *torT* transcription and identified the promoters regulated by IscR. Combining promoter mutational analysis with in vitro (DNase I footprinting and transcription assays) and in vivo reporter measurements, we defined the promoter elements that drive *torS* and *torT* transcription and quantified their relative contributions to overall *torS/torT* expression. We show that the −10 element of one *torS* promoter overlaps the −10 element of the divergently transcribed *torT* promoter and that IscR represses transcription from both promoters by binding to a site overlapping the shared −10 elements, occluding RNAP‐σ^70^ binding. Moreover, we show that strengthening this overlapping −10 region modestly increases *torS/torT* transcription and eliminates variability in *torCAD* expression during aerobic growth, even though the variant promoter remains repressible by IscR. Together, these findings indicate that both low basal promoter activity and transcriptional repression are essential for maintaining the TorS/TorT expression noise that underlies this bet‐hedging strategy, enabling *E. coli* to appropriately respond to abrupt decreases in O₂ availability.

## 2 Results

### 2.1 Defining the Promoter Elements Driving torS Transcription

Previously, two transcription start sites (designated P1*torS* TSS and P2*torS* TSS in Figure 1B) were mapped 42 bp apart upstream of *torS* by primer extension (Carey et al. 2019). Notably, the downstream TSS lies at the distal end of a HK022 *attB* site, indicating that its promoter (P2*torS*) is embedded within this bacteriophage attachment site. Within this region we identified putative −10 and −35 elements for P2*torS*, each with 4 out of 6 matches to consensus sequences (note matches on Figure 1B). Further upstream we identified corresponding −10 and −35 elements for P1*torS*, with a 4/6 match to the −10 consensus and a 3/6 match to the −35 consensus sequence (Figure 1B). The functions of the predicted promoters were assayed in vitro by comparing wild‐type promoter templates to those containing mutations of position A^−11^ of each −10 hexamer. This position was chosen to mutate because of its high conservation among −10 elements of *E. coli* σ^70^‐dependent promoters and its key role in base‐specific interaction with σ^70^ (Shultzaberger et al. 2007; Feklistov and Darst 2011). As observed previously (Carey et al. 2019), RNAP‐σ^70^ produced transcripts from both P1*torS* and P2*torS* (Figure 2A; lane 1) using supercoiled plasmid DNA harboring the wild‐type sequence from −152 to +28 bp relative to the TorS start codon. Transcript levels from P1*torS* were ~3‐fold higher than those from P2*torS* after normalization of transcript amounts to that of the RNA‐1 control, also encoded on this plasmid (Figure 2B; compare circle and square at 0 nM IscR). Importantly, our results demonstrate that an A^−11^T mutation in each promoter was sufficient to eliminate activity of the relevant promoter (Figure 2A; compare lanes 6 and 11 with lane 1), thereby confirming assignment of the −10 promoter elements. In addition, there was some evidence for steric hindrance of the downstream P2*torS* in our in vitro assay since an increase in P2*torS* activity occurred when the upstream P1*torS* was inactivated by the A^−11^T mutation (Figure 2A lanes 6–10; Figure 2B green triangles). In contrast, transcript levels from P1*torS* were similar regardless of whether P2*torS* was inactivated by the A^−11^T mutation (Figure 2B black circles and blue upside‐down triangles).

**FIGURE 2:**
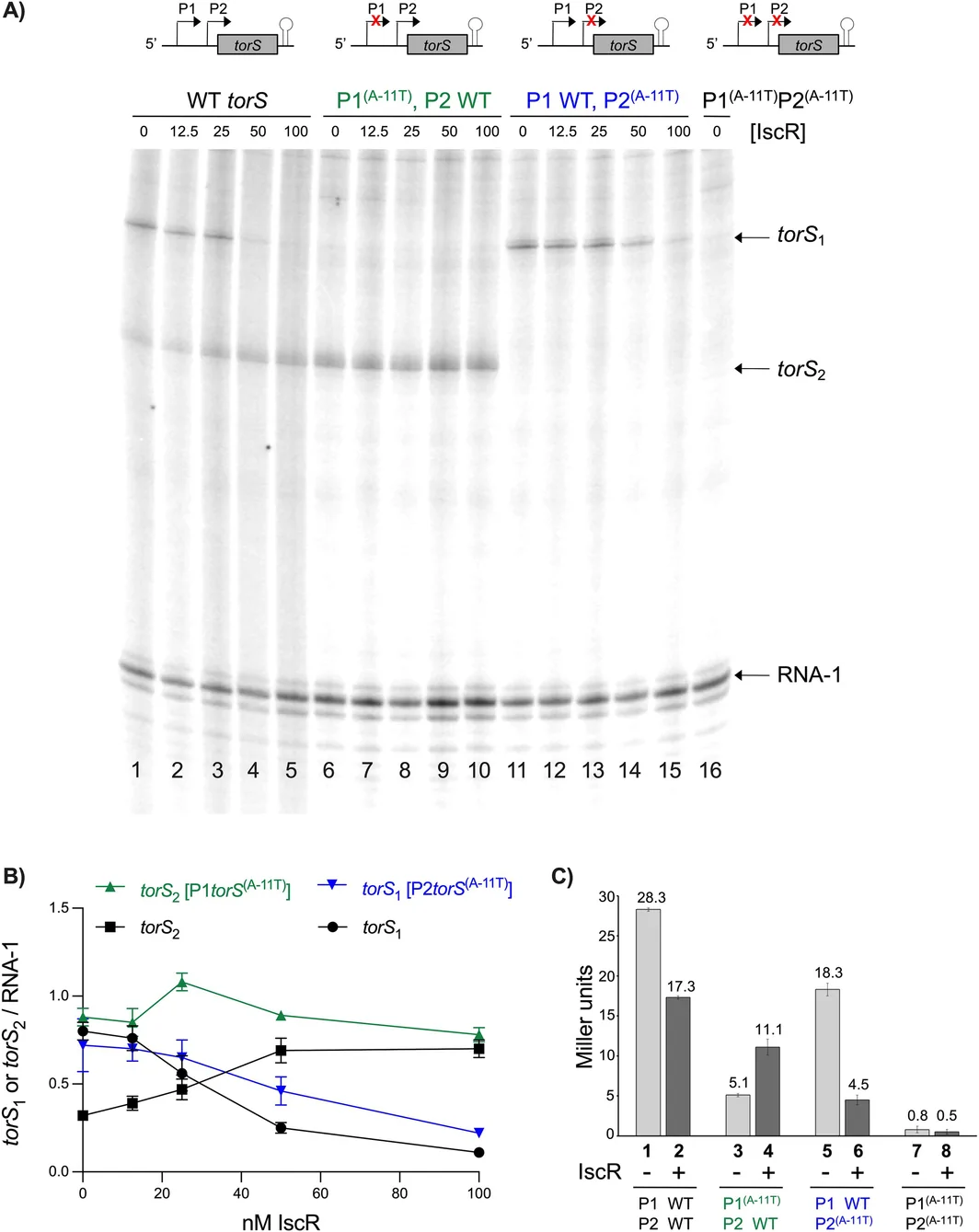
Comparison of P1*torS* and P2*torS* activities in vitro and in vivo. (A) In vitro transcription reaction products produced from P1*torS* and P2*torS* promoter variant plasmid templates diagrammed above the gel image. Transcripts produced from these plasmids are labeled on the right of the gel and the plasmid templates correspond to pPK12669, wild‐type (WT) P1*torS* and WT P2*torS* (lanes 1–5); pPK13105, P1*torS* ^(A‐11T)^ and WT P2*torS* (lanes 6–10); pPK13106, WT P1*torS* and P2*torS* ^(A‐11T)^ (lanes 11–15); and pPK13128, P1*torS* ^(A‐11T)^ and P2*torS* ^(A‐11T)^ (lane 16). Indicated concentrations (nM) of IscR were incubated with DNA for 30 min at 37°C prior to the addition of RNAP‐σ^70^ (to 50 nM) and subsequent incubation for 5 min. (B) Quantification of in vitro transcripts from results in panel A (lanes 1–15), along with technical replicates, normalized to the control RNA‐1 transcript and plotted as a function of IscR concentration. Transcripts labeled *torS*_1_ (black circles) and *torS*_2_ (black squares) were derived from pPK12669 containing WT P1*torS* and WT P2*torS*; the transcript labeled *torS*_2_ [P1*torS* ^(A‐11T)^] (green triangles) was derived from pPK13105 containing P1*torS* ^(A‐11T)^ and WT P2*torS*; and the transcript labeled *torS*_1_ [P2*torS* ^(^^A‐11T^^)^] (blue upside‐down triangles) was derived from pPK13106 containing WT P1*torS* and P2*torS* ^(A‐11T)^. Transcript levels from P2*torS* were multiplied by 1.25 to account for differences in the number of radiolabeled UTPs incorporated into *torS*_1_ and *torS*_2_ transcripts. (C) In vivo activity of P1*tor*S and P2*tor*S. β‐galactosidase activity, reported as Miller units, measured in strains containing a chromosomal promoter‐*lacZ* transcriptional fusion in which *lacZ* expression was driven by the sequence upstream of *torS* encompassing P1 and P2, or the A^−11^T mutant variants, identical to those described above. All strains were grown aerobically in the presence of TMAO. IscR^−^ strains are indicated with the light gray bars 1, 3, 5, 7 and IscR^+^ strains are dark gray bars 2, 4, 6, 8. The Miller units measured are indicated above each bar. All pairwise comparisons made were significant, with *p* values of < 0.0001 (one‐way ANOVA with Tukey's test).

To determine the relative contribution of each promoter to in vivo *torS* expression, we used the same DNA fragment from the in vitro transcription assays with and without the A^−11^T promoter variants to drive transcription of chromosomally encoded *lacZ* and assayed β‐galactosidase activity. To measure basal activity of these promoters, the constructs were assayed in strains lacking the repressor IscR. Expression from the wild‐type fragment containing both promoters (28.3 units) was just slightly higher than the combined expression from fragments containing only P2*torS* (with P1*torS* mutated; 5.1 units) or only P1*torS* (with P2*torS* mutated; 18.3 units) (Figure 2C; compare bars 1, 3, and 5). Additionally, expression driven by P1*torS* was approximately 3‐fold greater than that of P2*torS* (Figure 2C; compare bars 3 and 5), consistent with our in vitro findings. When both promoters were mutated, activity appeared to be abolished (Figure 2C; bar 7). Indeed, when grown on the same day, β‐galactosidase activity measured in the P1^(^^A‐11T^^)^P2^(^^A‐11T^^)^ *torS*‐*lacZ* reporter strain (2.2 ± 0.4 Miller units) was 40% of that measured in a control strain lacking any promoter DNA recombined upstream of chromosomal *lacZ* (5.3 ± 0.2 Miller units). Together, these in vitro and in vivo results indicate that basal levels of *torS* transcription are driven mostly from P1*torS*.

### 2.2 IscR Represses Transcription From P1torS but Not P2torS

We next examined whether both P1*torS* and P2*torS* activities are subject to IscR‐mediated repression using in vitro transcription assays. Increasing concentrations of IscR selectively repressed transcription from P1*torS*, regardless of whether P2*torS* was wild‐type or inactivated by the A^−11^T substitution, with ≥ 3‐fold repression at the highest IscR concentration tested (Figure 2A lanes 1–5 and 11–15; Figure 2B black circles and blue upside‐down triangles). In contrast, IscR had no effect on P2*torS* when P1*torS* was inactivated by A^−11^T (Figure 2A lanes 6–10; Figure 2B green triangles). This mutation at the distal end of the IscR type 2 binding site is not expected to perturb IscR binding since this substitution conserves the minor groove AT tract feature that binds the wing motif of IscR (Rajagopalan et al. 2013). When both promoters were intact, IscR addition led to an *increase* in transcription from P2*torS*, apparently as a consequence of IscR‐dependent repression of upstream P1*torS* (Figure 2A lanes 1–5; Figure 2B black squares). Because these promoters are in close proximity, we propose that transcription from P1*torS* sterically hinders RNAP‐σ^70^ binding at P2*torS*. Consequently, P2*torS* transcription increases when P1*torS* is rendered inactive either by IscR repression or mutation.

The effect of IscR on expression of these promoter variants was also examined in vivo using the *lacZ* reporter constructs. As observed in vitro, P1*torS* was repressed by IscR since β‐galactosidase activity was 4‐fold lower in the IscR^+^ strain compared to the IscR^−^ strain in which P1*torS* was intact and P2*torS* was inactivated by the A^−11^T mutation (Figure 2C; compare bars 5 and 6). In contrast, P2*torS* (P1^(A‐11T)^, P2 WT) activity appeared to increase ~2‐fold when IscR was present (Figure 2C; compare bars 3 and 4) even though our in vitro results suggest that IscR does not directly activate transcription of P2*torS* (Figure 2A lanes 6–10; Figure 2B green triangles). Although this was not investigated further, it is possible that any residual transcription from P1*torS* ^(A‐11T)^ in vivo might be repressed by IscR and relieve steric hindrance of RNAP‐σ^70^ binding to P2*torS*. The net result of this regulation is that IscR reduces overall transcriptional output of P*torS* 1.6‐fold (Figure 2C; compare bars 1 and 2) and this occurs by apparently shifting transcription from primarily the upstream P1*torS* to the downstream P2*torS* (Figure 2C; compare bars 4 vs. 6 and 3 vs. 5). The effect of IscR on overall *torS* repression was even stronger (4‐fold IscR dependent repression) in transcriptional fusion strains where *lacZ* was inserted after the native *torS* gene (Figure S1) in contrast to the strains used in these experiments in which the *torS* promoter region replaced the native *lac* promoter upstream of *lacZ*, suggesting possible additional genome context effects or posttranscriptional regulation.

### 2.3 P1torS and PtorT Have Overlapping −10 RNAP‐σ^70^ Recognition Elements

To characterize the divergent *torT* promoter, we first performed primer extension using RNA synthesized in vitro to map the *torT* + 1 TSS. A major transcript whose 5′ end mapped to a T, 18 nt upstream of the P1*torS* TSS was identified on the opposite strand (Figure S2), in agreement with a previous genome‐wide RNA profiling study (Ju et al. 2019). This location suggests that the putative −10 RNAP‐σ^70^ recognition element for P*torT* overlaps 5 out of 6 nucleotides that serve as the −10 element of P1*torS* on the opposite strand (Figure 1B). Furthermore, the predicted IscR binding site overlaps both the *torT* TSS and the shared −10 element, consistent with previous results that IscR represses expression of P*torT* in vivo (Carey et al. 2018).

The close proximity of the two TSSs and the overlapping −10 elements suggests that P1*torS* and P*torT* belong to the class of relatively understudied bidirectional promoters, which produce ~19% of all RNAs in *E. coli* (Warman et al. 2021). These bidirectional promoters permit RNA polymerase binding in either orientation, but only one orientation can be occupied at a time. To investigate whether mutation of the shared −10 element affects transcription in both directions, we examined P*torT* function by reversing the orientation of the P1*torS* ^(A‐11T)^ fragment. In this orientation, the T^−7^ position corresponds to A^−11^ on the opposite strand (Figure 1B). Because T^−7^, like A^−11^, is predicted to make base‐specific interaction with σ^70^ (Feklistov and Darst 2011), we hypothesized that changing T^−7^ to an A would disrupt P*torT* activity. To compare wild‐type P*torT* and P*torT* ^(T‐7A)^ activities, we used in vivo reporter assays rather than in vitro transcription assays due to the very low *torT* transcript levels produced from wild‐type P*torT* in vitro (Figure S3). The same promoter fragments used to assay P1*torS* and P1*torS* ^(A‐11T)^ (−140 to +40 bp relative to the *torT* start codon) were cloned in the opposite orientation upstream of *lacZ*. In a strain lacking IscR, activity of the P*torT* ^(T‐7A)^‐*lacZ* variant was nearly 14‐fold lower than that of wild‐type P*torT*‐*lacZ* (Figure 3; compare IscR^−^ activity from fragments 1 and 2), demonstrating that the T^−7^A mutation indeed disrupts P*torT* function. Taken together, these results show that a single nucleotide substitution in the shared −10 element impedes transcription from both P*torT* and P1*torS*, consistent with their classification of a bidirectional promoter pair. In contrast, mutation of the P2*torS* −10 element had no effect on transcription from P*torT* (Figure S4A; compare fragments 1 and 8). Therefore, all remaining constructs designed to investigate the bidirectional promoters and their regulation by IscR contained the P2*torS* ^(A‐11T)^ mutation to simplify interpretation.

**FIGURE 3:**
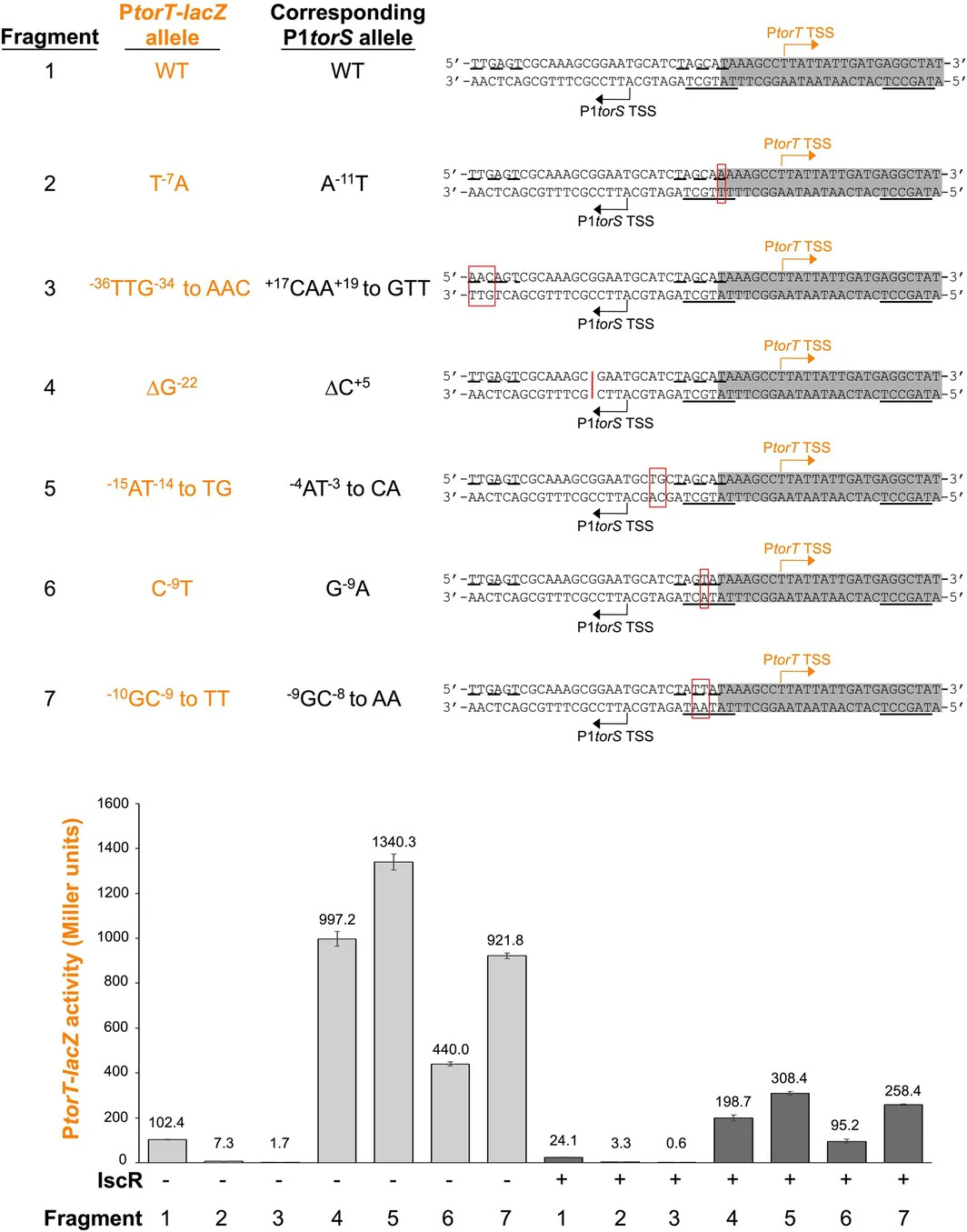
Mutational analysis of P*torT* activity in vivo. Promoter variants analyzed are indicated by the fragments depicted in top panel. Partial sequence (−36 to +19 bp relative to TSS of P*torT*) of the 180 bp P*torT* fragment driving expression of *lacZ* on the chromosome are shown. With exception of fragments 1 and 2, P2*torS* was inactivated in all fragments [P2*torS* ^(A‐11T)^]. The orange arrow marks the *torT* TSS of the promoter driving *lacZ* expression, mutations are boxed in red, and the IscR binding site is boxed in gray. The −10 and −35 RNAP‐σ^70^ recognition elements are underlined for P1*torS* (single) and P*torT* (dotted). Cultures were grown under aerobic conditions with TMAO and β‐galactosidase activity (Miller units) was measured from variants in IscR^−^ (light gray bars) and IscR^+^ (dark gray bars) strains. The Miller units measured are indicated above each bar and the relevant fragment assayed is listed under the bar. All pairwise comparisons made were significant to a *p* value of at least < 0.05 except comparisons in the IscR+ strains between fragments 1 and 2 and between fragments 1 and 3, which were not significant (one‐way ANOVA with Šidák's test).

We next examined the effect of disrupting the individual −35 RNAP‐σ^70^ recognition elements for each promoter. Inactivating P*torT* by mutation of its −35 element (^−36^TTG^−34^ to AAC) reduced *torT* expression (Figure 3; compare IscR‐ activity from fragments 1 and 3) but had little impact on transcription from P1*torS* (Figure S4B; compare fragments 8 and 3). Likewise, disrupting the −35 element of P1*torS* (^−35^TAG^−33^ to AAC) reduced *torS* expression ~5‐fold (Figure 4; compare IscR^**−**^ activity from fragments 8 and 9), while decreasing *torT* expression by less than 2‐fold (Figure S4A; compare fragments 8 and 9). Thus, although the −10 elements are shared between the two divergent promoters, the −35 elements function mostly independently.

**FIGURE 4:**
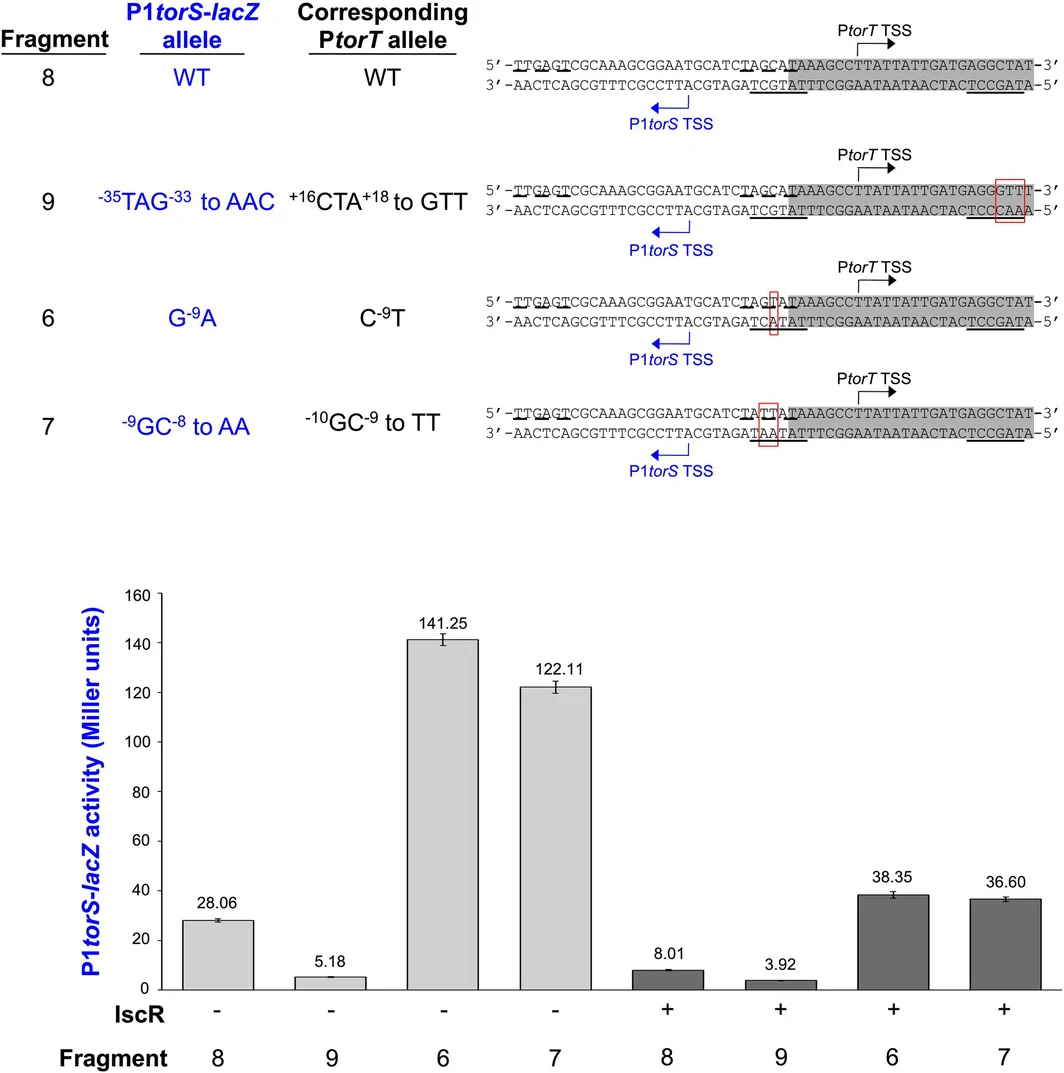
Mutational analysis of P1*torS* activity in vivo. Promoter variants analyzed are indicated by the fragments depicted in top panel. Partial sequence (−36 to +19 bp relative to TSS of P1*torS*) of the 180 bp P1*torS* fragment driving expression of *lacZ* on the chromosome are shown. P2*torS* was inactivated in all fragments [P2*torS* ^(A‐11T)^]. The blue arrow marks the *torS* TSS of the promoter driving *lacZ* expression, mutations are boxed in red, and the IscR binding site is boxed in gray. The −10 and −35 RNAP‐σ^70^ recognition elements are underlined for P1*torS* (single) and P*torT* (dotted). Cultures were grown under aerobic conditions with TMAO and β‐galactosidase activity (Miller units) was measured from variants in IscR^−^ (light gray bars) and IscR^+^ (dark gray bars) strains. The Miller units measured are indicated above each bar and the relevant fragment assayed is listed under the bar. All pairwise comparisons made were significant to a *p* value of at least < 0.05 except comparison in the IscR+ strains between fragments 8 and 9, which was not significant (one‐way ANOVA with Šidák's test).

We also introduced mutations predicted to enhance promoter function. Substitutions that improve the shared −10 element of P1*torS* towards consensus (5′TATAAT3′) were tested for their impact on basal expression of P*torT*‐*lacZ* (Figure 3) and P1*torS*‐*lacZ* (Figure 4) in strains lacking IscR; these substitutions included P1*torS* ^(G−9A)^ [equivalent to P*torT* ^(C−9T)^; fragment 6] and P1*torS* ^(−9GC−8 to AA)^ [equivalent to P*torT* ^(−10GC−9 to TT)^; fragment 7]. Importantly, both substitutions increased activity of each divergent promoter by 4‐fold or more. In contrast, mutations designed to optimize features expected to be unique to P*torT* had promoter‐specific effects. Improving −10 and −35 promoter element spacing to 17 bp [P*torT* ^(ΔG‐22)^; fragment 4] or creating an extended −10 element [P*torT* ^(−15AT−14 to TG)^; fragment 5] increased P*tor*T promoter activity 12–15‐fold in vivo (Figure 3; compare fragments 1, 4, and 5) and 3–5‐fold in vitro (Figure S3) with no detectable effect on P1*torS* activity (Figure S4B; compare fragments 8, 4, and 5). The improved promoters were also 4–5‐fold repressed by IscR similar to the native promoter (Figure 3; compare IscR‐ and IscR+ bars for fragments 1, 4–7). Together these results show that strengthening the shared −10 element increases transcription from both P1*torS* and P*torT*, whereas improvements to nonoverlapping features of P*torT* selectively enhance only its activity.

### 2.4 A Small Increase in Promoter Activity Eliminates Bet‐Hedging

The previously observed cell‐to‐cell variability in *torCAD* expression in aerobically grown populations has been hypothesized to depend on low transcription levels of *torS* and *torT*, due in part to IscR‐mediated repression (Carey et al. 2018). Our in vivo and in vitro studies suggest that even in the absence of IscR, P1*torS* and P*torT* are relatively weak promoters. This observation raised the possibility that low basal promoter strength, along with IscR repression, contributes to bet‐hedging.

We tested this possibility using the variant with a strengthened shared −10 element [P*torT* ^(C−9T)^, equivalent to P1*torS* ^(G−9A)^]. These strengthened variants exhibit ≥ 4‐fold higher activity compared to the wild‐type promoters and IscR repressed transcription from P*torT* ^(C−9T)^ and P1*torS* ^(G−9A)^ by a similar ≥ 4‐fold magnitude as the wildtype promoters (Figure 3, compare IscR‐ and IscR+ bars for fragments 1 and 6; Figure 4, compare IscR− and IscR+ bars for fragments 8 and 6). To determine whether this 4‐fold increase in promoter strength was sufficient to impact bet‐hedging, strains containing a fluorescent protein reporter of *torCAD* transcription and either native P*torT* or the P*torT* ^(C−9T)^ variant were constructed. These strains were grown aerobically in the presence of TMAO and transcription was measured in single cells by fluorescence microscopy. This fluorescence measurement was then correlated with cell growth following a transition to anaerobiosis. As observed previously for wild‐type P*torT* (Carey et al. 2018), *torCAD* expression was variable from cell‐to‐cell and only the subpopulation with high expression continued growing after O_2_ depletion. In contrast, cells harboring P*torT* ^(C−9T)^ uniformly exhibited high *torCAD* expression and nearly all these cells continued growing following the aerobic to anaerobic transition (Figure 5). These data suggest that bet‐hedging is lost when P*torT* and P1*torS* are simultaneously strengthened, presumably because even in the presence of IscR, increased expression of *torT* and *torS* likely leads to decreased cell‐to‐cell variation of TorT to TorS, reducing stochastic fluctuations in the TorS/TorT ratio, which in turn leads to fewer cells with low TorR phosphorylation. Thus, basal promoter strength is also a critical feature for *E. coli* to employ an O_2_‐dependent bet‐hedging strategy as a regulatory mechanism for TMAO respiration.

**FIGURE 5:**
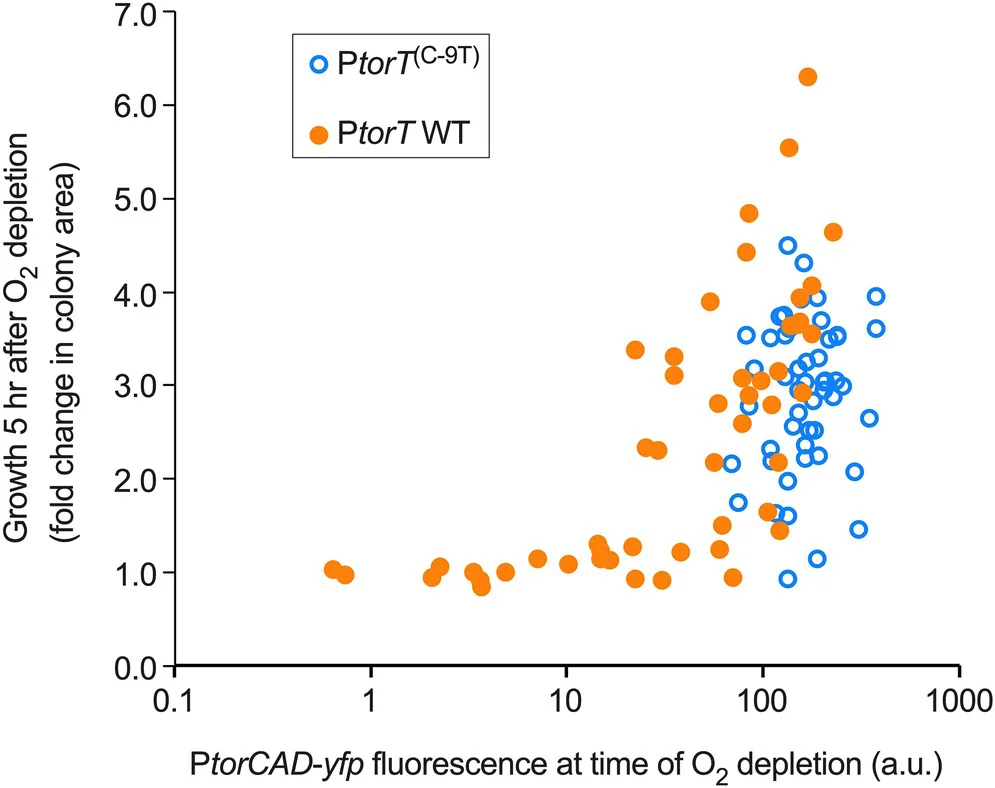
A base substitution in the overlapping −10 elements that simultaneously strengthens P*torT* and P1*torS* suppresses variability in *torCAD* expression under aerobic conditions. Cell growth after O_2_ depletion was measured and plotted as a function of P*torCAD*‐*yfp* fluorescence measured at the time of depletion. Each circle represents a microcolony of the P*torCAD*‐*yfp* strain harboring either wild‐type P*torT* (orange filled circles) or the P*torT* ^(C−9T)^ variant (blue open circles). Growth is quantified as the ratio of microcolony area 5 h after O_2_ depletion to the area of the parent cell at the time of depletion; a ratio of 1 indicates the cell has not grown. a.u., arbitrary units.

### 2.5 IscR Represses torS/torT Transcription by Occluding RNAP‐σ^70^ Promoter Recognition Elements

The IscR type 2 binding motif in the *torS*/*torT* intergenic region (Carey et al. 2018) is predicted to partially encompass the overlapping −10 elements, the *torT* TSS, and the P1*torS* −35 element (Figure 1B). The boundaries of the binding site were determined by DNase I footprinting experiments using a DNA fragment in which the 3′ end of the bottom strand was radiolabeled. IscR protected a region that encompasses the predicted type 2 motif and spans −8 to +21 bp relative to the *torT* TSS, and −38 to −10 bp relative to the upstream *torS* TSS (Figure 6; compare lanes 1 and 3), in agreement with the size of other DNase I footprints (Giel et al. 2006) and the target site contacts observed in the X‐ray crystal structure of IscR bound to a type 2 DNA site (Rajagopalan et al. 2013). Based on the location of the site, we hypothesized that through binding its recognition motif, IscR simultaneously occludes promoter recognition by RNAP‐σ^70^ for both P1*torS* and P*torT*. This was tested by comparing the RNAP‐σ^70^ footprints in the presence and absence of IscR using variants containing the −10 mutation to inactivate P2*torS* [P2*torS* ^(A‐11T)^] to simplify the analysis. In some cases, improved promoter elements were also used to overcome the weak binding of RNAP‐σ^70^ to the native promoters, especially P1*torS* (Figure S5; compare lanes 7 and 8). This weak binding affinity is consistent with the low basal activities measured for wild‐type P*torT* and P1*torS* in vivo (Figures 3 and 4). Using a fragment with an improved −10 element for P1*torS* and a −35 mutation to inactivate P*torT*, RNAP‐σ^70^ protected a region within P1*torS* extending from −40 to +19 bp relative to the upstream *torS* TSS (Figure 6; compare lanes 9 and 10 for variant P1*torS* ^(−9GC‐8 to AA/+17CAA+19 to GTT)^). Using a fragment with an extended −10 element for P*torT*, the RNAP‐σ^70^ footprint encompassed protection from −45 to +14 bp relative to the *torT* TSS and a major hypersensitive site at position −25 was apparent (Figure 6; compare lanes 5 and 6 for variant P*torT* ^(−15AT−14 to TG)^). The latter may be the result of RNAP‐σ^70^ constraining the DNA upon binding due to the longer spacer between the P*torT* −10 and −35 elements (18 bp versus the optimal 17 bp) (Hawley and Mcclure 1983). Similar areas of protection were observed for P1*torS* and P*torT* when the 3′ end of the top strand of the relevant DNA fragment was radiolabeled (Figure S6). Additionally, the protection pattern for either promoter is consistent with predictions of an RNAP‐promoter open complex (Ozoline and Tsyganov 1995). When reactions were performed in the presence of both IscR and RNAP‐σ^70^, however, the resulting footprints mirrored the footprint produced from IscR alone (Figures 6 and S6). This was the case for both the wild‐type promoter fragment and the improved promoter variants in which RNAP‐σ^70^ bound with higher affinity. These data imply that IscR can outcompete RNAP‐σ^70^ in binding to the *torS*/*torT* intergenic region and simultaneously repress transcription of both genes.

**FIGURE 6:**
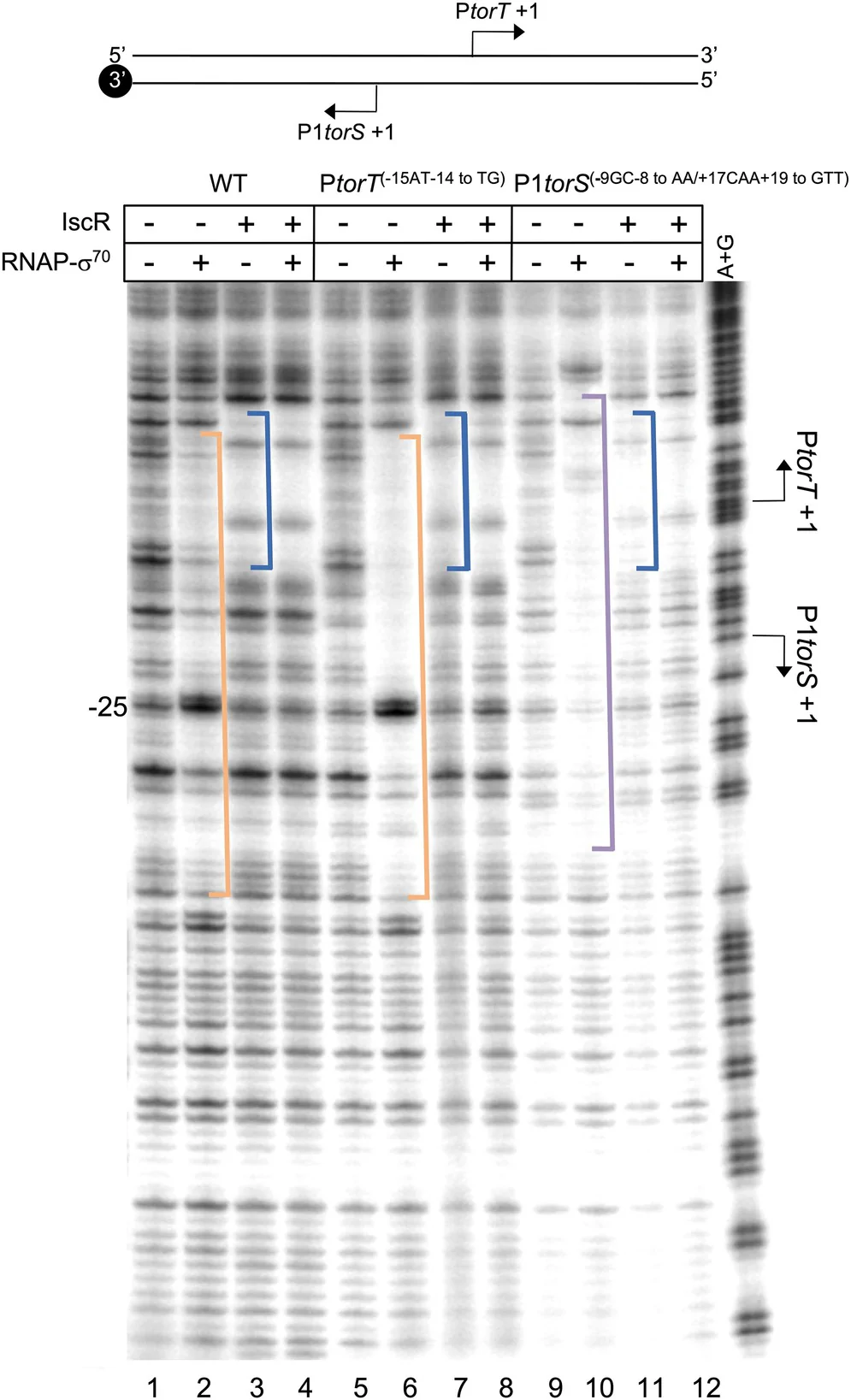
DNase I footprinting shows IscR binding to either P*torT* or P1*torS* prevents promoter recognition by RNAP‐σ^70^. The general scheme of the radiolabeled bottom strand fragment used in the footprinting assays is shown in the top diagram. DNA fragments assayed were isolated from plasmids pPK13116 [wild‐type P*torT* and P1*torS*; lanes 1–4], pPK13248 [strengthened P*torT* containing an extended −10 element; ^−15^AT^−14^ to TG; lanes 5–8], and pPK13258 [strengthened P1*torS* with improved −10 element and disrupted P*torT* −35 element, corresponding to P*torT* ^(−36TTG‐34 to AAC/−10GC−9 to TT)^; lanes 9–12]. All fragments contained the P2*torS* ^(A‐11T)^ mutation. The presence of IscR or RNAP‐σ^70^ in the reaction is indicated by the + symbol. Orange and purple lines mark the regions of protection by RNAP‐σ^70^ in P*torT* and P1*torS*, respectively; the blue lines mark the region protected by IscR. Position of DNase I enhancement is noted at position −25 relative to the P*torT* TSS. Samples were electrophoresed with a Maxam‐Gilbert (A + G) ladder.

### 2.6 Transcriptional Repression of torS/torT Requires Increased Levels of Apo‐IscR Under Aerobic Conditions

IscR‐mediated repression of *torS*/*torT* was observed primarily under aerobic growth conditions (Carey et al. 2018), presumably due to the ~10‐fold higher IscR protein levels present in aerobic cells compared to anaerobic cells (Giel et al. 2013). Additionally, the IscR binding motif in the *torS*/*torT* intergenic region was first identified as a type 2 motif (Carey et al. 2018), and was confirmed here. Other type 2 motif promoters (e.g., P*hyaA* and P*ydiU*) are primarily regulated in vivo by IscR apoprotein (apo‐IscR) (Nesbit et al. 2012; Chowdhury et al. 2021), which is the predominant protein form under aerobic conditions. In contrast, regulation of type 1 IscR binding motifs requires [2Fe‐2S]‐IscR, present predominantly under anaerobic conditions (Giel et al. 2013). To evaluate how protein levels and [2Fe‐2S] cluster occupancy affect *torS*/*torT* expression, we measured β‐galactosidase activity from promoter‐*lacZ* reporters in strains expressing wild‐type *iscR* or the apoprotein variant *iscR(C92A)* from IPTG‐inducible P*tac*. While promoter activity decreased in response to increasing IPTG concentrations under both aerobic and anaerobic growth conditions, we observed that wild‐type IscR was more efficient in repressing P1*torS* and P*torT* when O_2_ was present (Figure 7), suggesting that apo‐IscR is more efficient in repressing these promoters compared to [2Fe‐2S]‐IscR. Consistent with this notion, the amount of repression by the apoprotein variant IscR‐C92A at each IPTG concentration no longer depended on whether the cells were grown under aerobic versus anaerobic conditions (Figure 7). Western blot analysis demonstrated that aerobic levels of wild‐type IscR expressed from its native promoter correspond to levels of IscR‐C92A expressed from P*tac* induced with ~160 μM IPTG, whereas anaerobic levels of wild‐type IscR correspond to levels of IscR‐C92A expressed from P*tac* induced with ~20 μM IPTG (Figure S7). We observed little to no repression of P1*torS*/P*torT* by IscR‐C92A at 20 μM IPTG, whereas repression by IscR‐C92A at 160 μM IPTG was similar to that measured in an aerobically grown wild‐type strain (Figure 7). Therefore, the elevated levels of apo‐IscR present in aerobic cells are critical for the regulation of *torS* and *torT*, similar to that of other type 2 motif IscR regulated promoters.

**FIGURE 7:**
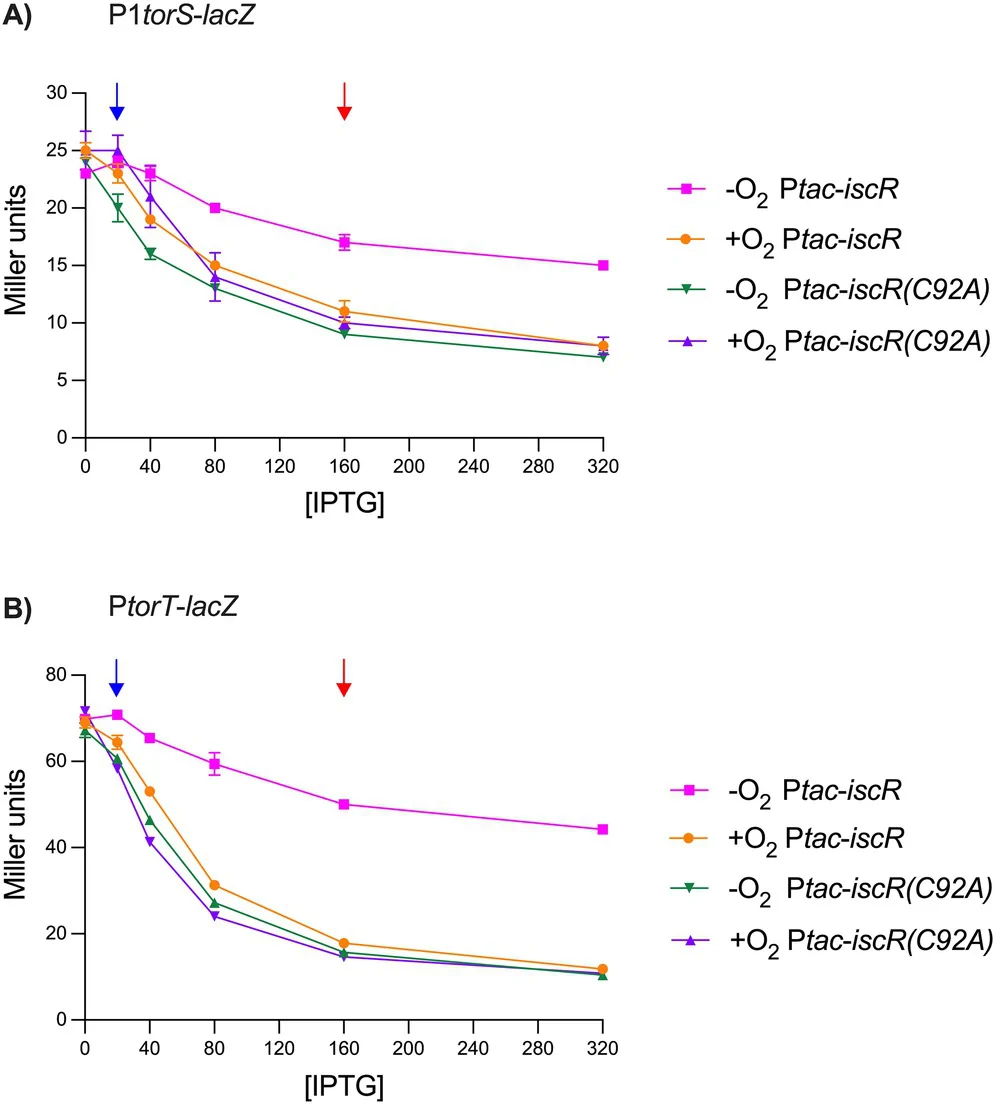
An increase in IscR abundance impacts transcriptional repression of *torS* and *torT*. β‐galactosidase activity was measured in strains containing either the P1*torS*‐*lacZ* variant in which P2*torS* was inactivated [P2*torS* ^(A‐11T)^] (A) or P*torT*‐*lacZ* (B) under aerobic and anaerobic growth conditions. Strains expressing chromosomally encoded *iscR* (pink squares and orange circles) or the apoprotein derivative *iscR(C92A)* (green and purple triangles) from P*tac* were grown in the presence of Tet to maintain a plasmid expressing LacI (pACYC*lacI* ^q^), and various concentrations of IPTG (μM). For comparison, the Miller units measured in P1*torS*‐*lacZ* strains expressing wild‐type chromosomal *iscR* from its native promoter or containing the Δ*iscR* allele were respectively 9 ± 0.4 and 21 ± 0.9 for aerobic conditions, and 22 ± 1.1 and 23 ± 0.3 for anaerobic conditions. The Miller units measured in P*torT*‐*lacZ* strains expressing wild‐type chromosomal *iscR* from its native promoter or containing the Δ*iscR* allele were respectively 13.6 ± 0.6 and 60.7 ± 0.6 for aerobic conditions, and 60.3 ± 0.8 and 63.9 ± 4.1 for anaerobic conditions. Arrows indicate the IPTG concentrations in which IscR‐C92A protein levels are similar to those of IscR in a wild‐type strain grown under aerobic (red) and anaerobic (blue) growth conditions as determined by Western blot analysis (Figure S7).

## 3 Discussion

This study addresses the transcriptional regulation of the TMAO sensor kinase subunits TorT and TorS. Prior work suggested that low expression of TorS and TorT generates noise in the TorS/TorT ratio, producing fluctuating amounts of TorR‐P, and ultimately, heterogeneous *torCAD* transcription during aerobic growth. Here we define how *torS* and *torT* are transcriptionally regulated and how this regulatory architecture supports an O_2_‐dependent bet‐hedging strategy in which only a subset of aerobic cells expresses TMAO reductase at levels sufficient for rapid adaptation upon O_2_ loss. However, our studies did not address whether additional mechanisms also impart noise in the TorS/TorT ratio, such as possible translational regulation and random partitioning of the low TorS and TorT levels between daughter cells upon cell division.

### 3.1 Bidirectional Promoter Architecture Governing torS and torT Expression

In vivo and in vitro characterization of the *torS*/*torT* intergenic region revealed three promoters (P1*torS*, P2*torS* and P*torT*) responsible for the divergent transcription of these genes. The upstream P1*torS* and the divergent P*torT* promoters function as a bidirectional promoter pair whose transcription start sites are separated by 18 bp—the most common spacing among bidirectional promoters in *E. coli* (Warman et al. 2021). Because these promoters share overlapping −10 elements, RNAP‐σ^70^ binding to one promoter can sterically occlude RNAP‐σ^70^ binding to the other, inherently limiting transcription initiation in either direction. However, recent findings by Warman et al. suggest that basal activity levels of bidirectional promoters studied thus far are likely too low to exhibit RNAP‐σ^70^ competition in vivo (Warman et al. 2026). Indeed, our analysis of basal P1*torS* and P*torT* activities indicates that this is also the case for this bidirectional promoter pair.

Rates of transcription initiation vary widely among promoters, and several intrinsic features contribute to the weak activity of P1*torS* and P*torT*. Both −10 elements deviate from the consensus (5′TATAAT3′), instead containing two G/C pairs that make DNA melting less favorable for RNAP‐σ^70^. A single A/T substitution in this shared region significantly enhanced RNAP‐σ^70^ binding and promoter activity, demonstrating that the suboptimal base composition of the shared −10 element is a key determinant of low expression. Neither promoter contains a strong consensus −35 element, and the highly conserved TTG motif—important for initial RNAP‐σ^70^ contacts—is TAG in P1*torS* (Haugen et al. 2008; Shultzaberger et al. 2007; Chen et al. 2020; Jensen and Galburt 2021). For P*torT*, the 18‐bp spacer between the −10 and −35 elements is also suboptimal; reducing it to 17 bp markedly improved expression. Together, these sequence features likely account for the low intrinsic activity of both promoters. Additionally, steps following RNAP‐σ^70^ binding may further limit P*torT* transcription, as suggested by its weaker in vitro transcript output despite relatively higher RNAP‐σ^70^ affinity than P1*torS*.

Our finding that the IscR binding site overlaps the shared −10 region and adjacent promoter elements explains how P1*torS* and P*torT* are both repressed by IscR. Indeed, DNase I footprinting demonstrated that IscR competitively blocks RNAP‐σ^70^ binding at both promoters, indicating that reduced RNAP recruitment is another mechanism enforcing low promoter activity under aerobic conditions.

### 3.2 Evolutionary Conservation and Implications for Bet‐Hedging

The *torS/torT* intergenic region is highly conserved across the *Enterobacteriaceae*, particularly the overlapping −10 and associated −35 elements and the IscR binding site (Carey et al. 2018). This conservation suggests selective pressure to maintain weak promoter activity for both genes. Enhancing the −10 element (as shown in this study) or disrupting the IscR site (Carey et al. 2018) each increases *torS*/*torT* transcription 2–4‐fold, levels sufficient to eliminate bet‐hedging by reducing noise in the TorS/TorT ratio and driving uniformly high TorR phosphorylation. These observations underscore the adaptive value of weak promoter activity and IscR repression in environments where TMAO is present.

The promoter elements of P2*torS*, in contrast, are embedded within the lambdoid phage HK022 *attB* site in many *E. coli* strains. In *E. coli* lysogenized by phage HK022, expression of *torS* is driven by an outward‐facing phage promoter, abolishing bet‐hedging and restricting *torCAD* expression to anaerobic conditions (Carey et al. 2019). This lysogen phenotype aligns with the behavior of typical anaerobic respiratory systems in *E. coli* (i.e., only expressed anaerobically) and highlights the functional consequences of native regulatory architecture in the absence of lysogenic phage.

Because P1*torS* overlaps with P*torT*, evolutionary constraints may have limited sequence changes to this tightly linked bidirectional promoter pair. By contrast, P2*torS* evolution may be independently constrained by unknown phage dynamics. Nevertheless, DNase I footprinting results showed that RNAP‐σ^70^ bound at P1*torS* occludes the P2*torS* −35 element, possibly explaining why transcription from P2*torS* increases when P1*torS* is repressed by IscR or is disrupted by mutation. This slight increase in P2*torS* activity in the presence of IscR when P1*torS* and P*torT* are repressed, in addition to IscR exhibiting a greater effect on in vivo repression of *torT* compared to *torS*, likely contributes noise to the TorS/TorT ratio under aerobic conditions by biasing synthesis of TorS over TorT.

### 3.3 O_2_ ‐Dependence of IscR Mediated Repression

IscR differentially controls transcription of genes depending upon its [2Fe‐2S] cluster ligation state: apo‐IscR regulates type 2 motif promoters, while [2Fe‐2S]‐IscR regulates type 1 motif promoters (Mettert and Kiley 2024). Furthermore, negative autoregulation of [2Fe‐2S]‐IscR and sensitivity of its [2Fe‐2S] cluster to oxidation by O_2_ and subsequent degradation by superoxide promote low [2Fe‐2S]‐IscR levels and high apo‐IscR levels in anaerobic and aerobic cells, respectively (Giel et al. 2013; Banerjee et al. 2025). Through varying the cellular concentration of IscR or its apoprotein variant (IscR‐C92A), we confirmed that elevated aerobic apo‐IscR protein levels are critical for repression of *torS*/*torT* via a type 2 motif. In contrast, low anaerobic [2Fe‐2S]‐IscR levels are not sufficient to inhibit *torS*/*torT* transcription, thus explaining why IscR‐mediated repression of these genes is limited to aerobic conditions. It is well known that apo‐IscR directly represses transcription of at least three operons encoding anaerobic respiratory enzymes [hydrogenase‐1 (*hyaABCDEF*), hydrogenase‐2 (*hybOABCDEFG*), and a periplasmic nitrate reductase (*napFDAGHBC*)] under aerobic conditions (Giel et al. 2006). Thus, IscR appears to have a broader role to limit expression of anaerobic respiratory enzymes during aerobic growth.

In summary, the findings presented here have broadened our understanding of how transcriptional regulation contributes to the bet‐hedging strategy for TMAO reductase expression in *E. coli* . We have provided evidence that both promoter strength and high levels of apo‐IscR contribute to noise in the TorT‐TorS‐TorR signaling cascade under aerobic conditions in the presence of TMAO. In turn, this noise creates heterogeneous expression of *torCAD*, enabling *E. coli* to efficiently transition to TMAO respiration upon the sudden loss of O_2_. Such a strategy illustrates how bacteria use promoter architecture and regulatory systems to tune noise levels to beneficial ranges.

## 4 Experimental Procedures

### 4.1 Strain and Plasmid Construction

Strains and plasmids used in this work are listed in Table 1, and sequences of primers used are available upon request. All genotypes were confirmed by DNA sequencing.

**TABLE 1:** Strains and plasmids used in this work^a^.

Strain or plasmid	Relevant genotype or phenotype	Reference or source
**Strains**		
MG1655	λ^−^ F^−^ *rph‐1*	Laboratory stock
PK9012	MG1655 Δ*mutS*::*Tn10* λ*c1857* Δ*cro‐bioA*	Beauchene et al. (2017)
PK11396	PK9012 (Δ99 bp)::FRT‐*cat*‐FRT‐*araC*‐P*bad*‐RBS‐*iscS*	This study
PK11440	PK11396 *bla*‐P*tac*‐*iscR*	This study
PK11439	PK11396 *bla*‐P*tac*‐*iscR(C92A)*	This study
PK4854	MG1655 Δ*iscR*	Schwartz et al. (2001)
PK12732	MG1655 Δ*iscR::cat*	This study
JNC166	MG1655 *lacZYA*::FRT *torS*‐*lacZ*‐FRT‐*kan*‐FRT	Carey et al. (2018)
PK13068	MG1655 *torS*(−152 to +28 bp relative to the TorS start codon)‐*lacZ* (in *lac* region)	This study
PK13069	PK4854 *torS*(−152 to +28 bp relative to the TorS start codon)‐*lacZ* (in *lac* region)	This study
PK13155	PK13068 but P1*torS* ^(A‐11T)^	This study
PK13156	PK13069 but P1*torS* ^(A‐11T)^	This study
PK13157	PK13068 but P2*torS* ^(A‐11T)^	This study
PK13158	PK13069 but P2*torS* ^(A‐11T)^	This study
PK13159	PK13068 but P1*torS* ^(A‐11T)^P2*torS* ^(A‐11T)^	This study
PK13160	PK13069 but P1*torS* ^(A‐11T)^P2*torS* ^(A‐11T)^	This study
PK13443	PK13157 with P1*torS* ^(−35TAG‐33 to AAC)^	This study
PK13219	PK13158 with P1*torS* ^(−35TAG‐33 to AAC)^	This study
PK13240	PK13257 with P1*torS* ^(G−9A)^	This study
PK13225	PK13158 with P1*torS* ^(G−9A)^	This study
PK13242	PK13157 with P1*torS* ^(−9GC‐8 to AA)^	This study
PK13227	PK13158 with P1*torS* ^(−9GC‐8 to AA)^	This study
PK13445	PK13157 with P1*torS* ^(+17CAA+19 to GTT)^	This study
PK13217	PK13158 with P1*torS* ^(+17CAA+19 to GTT)^	This study
PK13446	PK13157 with P1*torS* ^(ΔC+5)^	This study
PK13221	PK13158 with P1*torS* ^(ΔC+5)^	This study
PK13447	PK13157 with P1*torS* ^(4AT‐3 to CA)^	This study
PK13229	PK13158 with P1*torS* ^(−4AT‐3 to CA)^	This study
PK13334	PK13157 but Δ*lacY* + pACYC*lacI* ^q^	This study
PK13335	PK13334 but *bla*‐P*tac‐iscR‐*(Δ99 bp)::FRT‐*cat‐*FRT‐*araC*‐P*bad‐*RBS*‐iscS*	This study
PK13336	PK13334 but *bla*‐P*tac‐iscR(C92A)‐*(Δ99 bp)::FRT‐*cat‐*FRT‐*araC*‐P*bad‐*RBS*‐iscS*	This study
PK13070	MG1655 *torT*(−140 to +40 bp relative to the TorT start codon)‐*lacZ* (in *lac* region)	This study
PK13071	PK4854 *torT*(−140 to +40 bp relative to the TorT start codon)‐*lacZ* (in *lac* region)	This study
PK13161	PK13070 but P*torT* ^(T‐7A)^	This study
PK13162	PK13071 but P*torT* ^(T‐7A)^	This study
PK13163	PK13070 but P*torT* ^(T‐49A)^	This study
PK13164	PK13071 but P*torT* ^(T‐49A)^	This study
PK13442	PK13163 with P*torT* ^(−36TTG‐34 to AAC)^	This study
PK13216	PK13164 with P*torT* ^(−36TTG‐34 to AAC)^	This study
PK13238	PK13163 with P*torT* ^(ΔG‐22)^	This study
PK13220	PK13164 with P*torT* ^(ΔG‐22)^	This study
PK13243	PK13163 with P*torT* ^(15AT−14 to TG)^	This study
PK13228	PK13164 with P*torT* ^(−15AT−14 to TG)^	This study
PK13448	PK13163 with P*torT* ^(C−9T)^	This study
PK13224	PK13164 with P*torT* ^(C−9T)^	This study
PK13241	PK13163 with P*torT* ^(−10GC−9 to TT)^	This study
PK13226	PK13164 with P*torT* ^(−10GC−9 to TT)^	This study
PK13444	PK13163 with P*torT* ^(+16CTA+18 to GTT)^	This study
PK13218	PK13164 with P*torT* ^(+16CTA+18 to GTT)^	This study
PK13327	PK13070 but Δ*lacY* + pACYC*lacI* ^q^	This study
PK13321	PK13327 but *bla*‐P*tac‐iscR‐*(Δ99 bp)::FRT‐*cat‐*FRT*‐araC*‐P*bad‐*RBS*‐iscS*	This study
PK13324	PK13327 but *bla*‐P*tac‐iscR(C92A)‐*(Δ99 bp)::FRT‐*cat‐*FRT‐*araC*‐P*bad‐*RBS*‐iscS*	This study
MMR65	MG1655 *att*λ::(*cat* P*torCAD*‐*yfp*) Δ*xylAFG*::P*tetA*‐mcherry‐FRT	Carey et al. (2018)
JNC108	MG1655 (Δ‐41 to +72 bp relative to the TorT start codon)::(*cat* I‐SceI cut site)	Carey et al. (2018)
MMR130	MG1655 *att*λ::(FRT P*torCAD‐yfp*) *ompA*‐*cfp*	Roggiani and Goulian (2015)
JNC110	MG1655 *att*λ::(FRT P*torCAD*‐*yfp*) *ompA*‐*cfp* (Δ‐41 to +72 bp relative to the TorT start codon)::(*cat* I‐SceI cut site)	This study
PK13361	MG1655 *att*λ::(FRT *PtorCAD*‐*yfp*) *ompA*‐*cfp* P*torT* ^(C−9T)^	This study
**Plasmids**		
pKD46	ori(pSC101) *rep101*(ts)P*araB*‐*gam*‐*bet*‐*exo araC amp*	Datsenko and Wanner (2000)
pKD32	FRT‐*cat*‐FRT	B. L. Wanner
pACYC*lacI* ^q^	*lacI* ^q^ in pACYC184 Tet^r^	Boyd et al. (1987)
pPK7035	pBR322 with *kan* from pHP45Ω and BamHI–NdeI fragment from pRS1553	Kang et al. (2005)
pPK7179	pUC19‐*spf*' with XhoI site replacing SalI site, Ap^r^	Kang et al. (2005)
pPK12669	pPK7179 with −152 to +28 bp relative to the TorS start codon in XhoI/BamHI sites	Carey et al. (2019)
pPK13105	pPK12669 but P1*torS* ^(A‐11T)^	This study
pPK13106	pPK12669 but P2*torS* ^(A‐11T)^	This study
pPK13128	pPK12669 but P1*torS* ^(A‐11T)^P2*torS* ^(A‐11T)^	This study
pPK12347	pPK7179 with −140 to +40 bp relative to the TorT start codon in XhoI/BamHI sites	This study
pPK13116	pPK12347 but P*torT* ^(T‐49A)^	This study
pPK13244	pPK13116 with P*torT* ^(+16CTA+18 to GTT)^	This study
pPK13245	pPK13116 with P*torT* ^(−36TTG‐34 to AAC)^	This study
pPK13255	pPK13116 with P*torT* ^(−36TTG‐34 to AAC/+16CTA+18 to GTT)^	This study
pPK13246	pPK13116 with P*torT* ^(ΔG‐22)^	This study
pPK13248	pPK13116 with P*torT* ^(−15AT−14 to TG)^	This study
pPK13258	pPK13116 with P*torT* ^(−36TTG‐34 to AAC/−10GC−9 to TT)^	This study
pWRG100	ori(R6K) FRT‐*cat*‐FRT *amp* I‐SceI cut site	Blank et al. (2011)
pWRG99	pKD46 *tetR* P*tetA*‐*I‐SceI*	Blank et al. (2011)
pPK13344	pPK7035 with −140 to +160 bp relative to the TorT start codon in XhoI/BamHI sites	This study
pPK13349	pPK13344 but P*torT* ^(C−9T)^	This study
pPK9112	*iscRS* fragment in EcoRV site of pBR322	Giel et al. (2013)
pPK11385	pPK9112 with deletion encompassing 99 bp upstream of the IscS start codon (Δ99 bp)	This study
pPK9125	FRT‐*cat*‐FRT cloned in SphI site of pBAD/Myc‐hisC	Giel et al. (2013)
pPK11394	pPK11385 with FRT‐*cat*‐FRT‐*araC*‐P*bad*‐RBS cloned into EcoRI site upstream of *iscS*	This study
pPK9156	*bla*‐P*tac*‐*iscR* cloned into HindIII/BamHI sites of pACYC*lacI* ^q^	Nesbit et al. (2012)
pPK9157	pPK9156 but *bla*‐P*tac‐iscR(C92A)*	Nesbit et al. (2012)

^a^ Base pair positions are relative to the TSS for the promoter described unless otherwise indicated.

Plasmids for in vitro transcription assays and DNase I footprinting experiments were derived from pPK7179. The 180 bp promoter region for *torS* (−152 to +28 bp relative to the TorS start codon) and *torT* (−140 to +40 bp relative to the TorT start codon) was PCR amplified from MG1655 using primer sets with XhoI/BamHI sites, and the fragment was cloned in both orientations in the same sites of pPK7179, creating pPK12669 and pPK12347, respectively. Subsequent promoter mutations (Table 1) were made by site‐directed mutagenesis using PfuTurbo DNA polymerase (Agilent Technologies).

Chromosomal promoter‐*lacZ* reporter strains were constructed by first cloning the same promoter fragments described above into the XhoI/BamHI sites of pPK7035 and subsequently introducing promoter mutations by site directed mutagenesis as indicated in Table 1. Mutations made to the promoter elements were specifically designed to not perturb the IscR binding site in cases where repression was being investigated. Wild‐type and variant *lacI*‐*kan*‐promoter‐*lacZ* alleles from these plasmids were PCR amplified, recombined onto the chromosome in the *lac* promoter region of MG1655 containing pKD46 as previously described (Datsenko and Wanner 2000), and transduced into MG1655 and PK4854 using P1 *vir*. Using the same recombination method, a deletion of *iscR* in MG1655 containing pKD46 was constructed by replacing the coding region of *iscR* with *cat* from pKD32 to generate PK12732. This ∆*iscR*::*cat* allele was transduced by P1 *vir* into JNC166, a reporter strain with *lacZ* inserted downstream of the *torS* coding sequence on the MG1655 chromosome.

To evaluate the effects of wild‐type IscR or IscR‐C92A protein levels on P1*torS*‐*lacZ* and P*torT*‐*lacZ* expression, strains containing *iscR* or *iscR(C92A)* under control of P*tac* on the chromosome were constructed as previously described (Giel et al. 2013), except that the native RyhB small RNA target site within the *iscS* transcript was replaced with an engineered ribosomal binding site (RBS; 5′AGGAG3′). First, 99 bp containing the RyhB site located upstream of the IscS start codon was deleted from pPK9112 by site‐directed mutagenesis, resulting in pPK11385. Second, a DNA fragment encompassing FRT‐*cat*‐FRT‐*araC*‐P*bad*‐RBS was PCR amplified from pPK9125 using primers with EcoRI sites and cloned into the EcoRI site immediately upstream of *iscS* in pPK11385, creating pPK11394. Fragment FRT‐*cat*‐FRT‐*araC*‐P*bad*‐RBS‐*iscS* was PCR amplified from pPK11394 using primers with homologous ends such that when recombined onto the chromosome of PK9012, this fragment replaced the 99 bp containing the RyhB site upstream of the IscS start codon (Δ99 bp), generating PK11396. Fragments *bla*‐P*tac*‐*iscR* or *bla*‐P*tac*‐*iscR(C92A)* were PCR amplified from pPK9156 and pPK9157, respectively, and electroporated into PK11396 to create strains PK11440 and PK11439, respectively. Finally, P1 *vir* transduction was used to move the *bla*‐P*tac*‐*iscR*‐(Δ99 bp)::(FRT‐*cat*‐FRT‐*araC*‐P*bad*‐RBS‐*iscS*) allele [or the *iscR(C92A)* variant] into P1*torS*‐*lacZ* and P*torT*‐*lacZ* reporter strains that also lacked *lacY* and contained pACYC*lacI* ^q^ (PK13334 and PK13327, respectively), generating strains PK13335, PK13336, PK13321, and PK13324.

To introduce a point mutation within native P*torT* of a P*torCAD*‐*yfp* fluorescent reporter strain, we used a scarless mutagenesis technique as previously described (Carey et al. 2018; Blank et al. 2011). Briefly, a DNA fragment containing *cat* and an I‐SceI cut site was PCR amplified from pWRG100 using primers with homologous ends such that when recombined at the *tor* locus of MG1655 containing pKD46, it replaced −41 to +72 bp relative to the TorT start codon with *cat* and the I‐SceI cut site. After curing this strain of pKD46 (to create JNC108), the modified *tor* locus was moved into strain MMR130 by P1 *vir* transduction. Chloramphenicol‐resistant transductants were screened for CFP fluorescence, and a CFP‐positive colony was picked to create JNC110. JNC110 was transformed with pWRG99, a derivative of pKD46 that contains a tetracycline (Tet)‐inducible I‐SceI gene. A mutant *torT* promoter fragment (−140 to +160 bp relative to the TorT start codon) in which C at position −9 relative to the *torT* TSS was mutated to a T was PCR amplified from pPK13349 and recombined onto the chromosome of JNC110 containing pWRG99. Colonies with the correct genotype were selected by plating on 0.5 μg mL^−1^ anhydrotetracycline to induce expression of I‐SceI, which would inhibit growth of cells harboring the I‐SceI cut site but not those containing P*torT* ^(C−9T)^. The resulting strain (PK13361) was sensitive to chloramphenicol and ampicillin, and P*torT* ^(C−9T)^ was sequenced verified.

### 4.2 In Vitro Transcription Assays

Assays were carried out as described in Carey et al. (2019). Since prior in vitro experiments demonstrated that an IscR apoprotein variant (IscR‐C92A) is sufficient to bind type 2 sites, assays were performed with this protein form isolated as previously described (Nesbit et al. 2009; Giel et al. 2006). Where indicated, reaction mixtures were incubated with IscR for 30 min at 37°C prior to initiating transcription with the addition of RNAP‐σ^70^ (NEB) to a final concentration of 50 nM. Transcripts were quantified (Molecular Dynamics ImageQuant) and transcript levels from P2*torS* were multiplied by 1.25 to account for differences in the number of radiolabeled UTPs incorporated into *torS*_1_ and *torS*_2_ transcripts. Finally, levels of *torS/torT* transcripts were normalized to the amount of RNA‐1 transcript. Each assay was repeated at least three times.

### 4.3 Primer Extension Assays

Assays were carried out as described in Carey et al. (2019) except a ^32^P‐labeled primer designed to anneal to the RNA transcript downstream of the TorT start codon was used. This same primer was also used in DNA sequencing reactions performed with the Sequenase Version 2.0 DNA Sequencing Kit (USB).

### 4.4 DNase I Footprinting Experiments

DNA fragments were isolated from pPK13116 derivatives after digestion with HindIII/BamHI; radiolabeled at either the HindIII 3′ end (bottom strand) or the BamHI 3′ end (top strand) with [α‐^32^P]‐dATP or [α‐^32^P]‐dGTP (PerkinElmer), respectively, using Sequenase Version 2.0; isolated from a nondenaturing acrylamide gel and subsequently purified with elutip‐d columns (Schleicher and Schuell). Footprinting assays were performed by incubating final concentrations of ~5 nM labeled DNA with either 100 nM IscR, 100 nM RNAP‐σ^70^, or both for 30 min at 37°C in 40 mM Tris (pH 7.9), 30 mM KCl, 100 μg mL^−1^ BSA and 1 mM DTT, followed by the addition of heparin to 10 μg mL^−1^ for 10 s, and subsequent addition of DNase I to 0.8 μg mL^−1^ (Worthington) for 30 s. Reactions were terminated by addition of sodium acetate and EDTA (final concentrations of 300 nM and 20 nM, respectively); ethanol precipitated; resuspended in urea loading dye; heated for 60 s at 90°C; and loaded onto a 7 M urea, 8% polyacrylamide gel in 0.5× TBE buffer. An A + G ladder was made by formic acid modification of the radiolabeled DNA, followed by piperidine cleavage. Reaction products were visualized by phosphorimaging.

### 4.5 β‐Galactosidase Assays

For assays presented in Figures 2C, 3, 4, S1, and S4, cultures were grown aerobically by shaking (250 rpm) to an optical density at 600 nm (OD_600_) of 0.2 at 37°C in minimal A medium (Miller 1992), containing 0.2% glucose and 10 mM TMAO. For assays presented in Figure 7, cultures were grown aerobically by shaking or anaerobically in screw‐cap tubes to an OD_600_ of 0.1 to 0.2 in minimal A medium containing 20 mM arabinose, 0.2% casamino acids, and 10 mM TMAO. Where indicated, the medium was additionally supplemented with 10 μg mL^−1^ Tet and indicated concentrations of isopropyl‐β‐D‐thiogalactopyranoside (IPTG). For all assays, 100 μg mL^−1^ spectinomycin was added to culture samples to terminate further protein synthesis and cell growth, and cells were kept on ice until assayed for β‐galactosidase activity as previously described (Miller 1972). Assays were repeated at least three independent times, and error bars represent the standard errors of three biological replicates.

### 4.6 Western Blot Analysis

Strains were grown as described for β‐galactosidase assays presented in Figure 7. Culture aliquots (1 mL) were centrifuged, the supernatant was removed, and cell pellets were frozen at −80°C. Pellets were thawed, resuspended in SDS‐loading buffer, heated for 15 min at 90°C, and loaded onto a 15% SDS‐polyacrylamide gel for electrophoresis. Proteins were then transferred to a nitrocellulose membrane by Western transfer, and IscR levels were detected using α‐IscR rabbit primary antibodies that were precleared with lysate from strain PK4854 (Harlow and Lane 1988) and horseradish‐peroxidase‐conjugated goat anti‐rabbit secondary antibodies (Santa Cruz Biotechnology). After treatment of blots with ECL start Western blotting detection reagent (Amersham), chemiluminescence was quantified using an Azure cSeries instrument and ImageJ software. Western blots were performed three times with independently grown cultures.

### 4.7 Aerobic‐To‐Anaerobic Transition Microscopy

Microscopy was performed as described in Carey et al. (2018) except that no Δ*torC* control strain was included.

## Author Contributions

**Jeffrey N. Carey:** investigation, writing – review and editing. **Mark Goulian:** conceptualization, writing – review and editing, funding acquisition. **Wilma Ross:** conceptualization, writing – review and editing, formal analysis. **Erin L. Mettert:** conceptualization, investigation, writing – original draft, writing – review and editing, formal analysis. **Patricia J. Kiley:** conceptualization, writing – original draft, writing – review and editing, funding acquisition, formal analysis, supervision, project administration.

## Funding

This work was supported by the National Institutes of Health (R01GM115894 to P.J.K.; R01GM080279 and R35GM139541 to M. G.; and R01GM37048 to Richard L. Gourse).

## Ethics Statement

The authors have nothing to report.

## Conflicts of Interest

The authors declare no conflicts of interest.

## Supporting information


**Figure S1:** β‐galactosidase activity (Miller units) was measured in IscR^−^ (light gray bars) and IscR^+^ (dark gray bars) strains grown under aerobic conditions. Strains contained either a P*torS*‐*lacZ* transcriptional fusion in which the sequence upstream of *torS* encompassing P1 and P2 replaced the native *lac* promoter, or a transcription fusion in which *lacZ* was inserted after the native *torS* coding sequence on the chromosome. The Miller units measured are indicated above each bar.
**Figure S2:** Identification of the *torT* transcription start site. The *torT* TSS was determined by primer extension using RNA prepared in vitro in which supercoiled plasmid DNA (pPK12347) harboring the sequence upstream of *torT* encompassing −140 to +40 bp relative to the predicted TorT start codon served as template. The products produced from DNA sequencing of pPK12347 are shown in lanes 1–4, and primer extension reactions with the same radiolabeled primer are shown in lane 5. For reference, partial sequence of P*torT* is shown, with the arrow marking the major TSS identified in this experiment (T). The gel image was digitally altered to remove a lane that was originally between lanes 4 and 5.
**Figure S3:** Promoter mutation analysis for P*torT* was carried out using in vitro transcription assays. Serving as DNA templates were supercoiled plasmids harboring the control RNA‐1 promoter and either wild‐type P*torT* (−120 to +60 bp relative to the *torT* TSS) or variants containing the indicated mutations relative to the *torT* TSS. All templates contained the P2*torS* ^(A‐11T)^ mutation. Transcription products from each promoter are indicated. Templates included pPK13116 [wild‐type], pPK13244 [disrupted P1*torS* −35 element corresponding to P1*torS* ^(−35TAG‐33 to AAC)^], pPK13245 [disrupted P*torT* −35 element], pPK13255 [disrupted −35 elements in both P*torT* and P1*torS*], pPK13246 [strengthened P*torT* with 17 bp spacing between −10 and −35 elements], and pPK13248 [strengthened P*torT* containing an extended −10 element]. The gel image was digitally altered to remove a lane that was originally between those labeled ∆G^−22^ and ^−15^AT^−14^ to TG.
**Figure S4:** Promoter mutation analysis was carried out by measuring β‐galactosidase activity (Miller units) from variants of P*torT*‐*lacZ* (A) and P1*torS*‐*lacZ* (B) in IscR^−^ strains grown under aerobic conditions. Partial sequence of the 180 bp promoter fragment upstream of *lacZ* on the chromosome are shown (−60 to +19 bp relative to the P*torT* TSS; −36 to +43 bp relative to the P1*torS* TSS). With exception of fragment 1, P2*torS* was inactivated in all fragments [P2*torS* ^(A‐11T)^]. The orange and blue arrows mark the TSS of the promoter driving *lacZ* expression, mutations are boxed in red, and the IscR binding site is boxed in gray. The −10 and −35 RNAP‐σ^70^ recognition elements are underlined for P1*torS* (single), P2*torS* (double), and P*torT* (dotted).
**Figure S5:** RNAP‐σ^70^ provides little protection of P1*torS* from DNase I cleavage. The general scheme of the radiolabeled bottom strand fragment used in the footprinting assays is shown in the top diagram. DNA fragments assayed were isolated from plasmids pPK13116 [wild‐type P*torT*; lanes 1 and 2], pPK13244 [disrupted P1*torS* −35 element corresponding to P1*torS* ^(−35TAG‐33 to AAC)^; lanes 3 and 4], pPK13248 [strengthened P*torT* containing an extended −10 element; lanes 5 and 6], pPK13245 [disrupted P*torT* −35 element, corresponding to P*torT* ^(−36TTG‐34 to AAC)^; lanes 7 and 8], pPK13258 [strengthened P1*torS* with improved −10 element and disrupted P*torT* −35 element, corresponding to P*torT* ^(−36TTG‐34 to AAC/−10GC−9 to TT)^; lanes 9 and 10], and pPK13255 [disrupted −35 elements in both promoters, corresponding to P*torT* ^(−36TTG‐34 to AAC/+16CTA+18 to GTT)^; lanes 11 and 12]. All fragments contained the P2*torS* ^(A‐11T)^ mutation. The presence of RNAP‐σ^70^ in the reaction is indicated by the + symbol. Orange and purple lines mark the regions of protection by RNAP‐σ^70^ in P*torT* and P1*torS*, respectively. Position of DNase I enhancement is noted at position −25 relative to the P*torT* TSS. Samples were electrophoresed with a Maxam‐Gilbert (A + G) ladder.
**Figure S6:** IscR binding to either P*torT* or P1*torS* prevents promoter recognition by RNAP‐σ^70^. The general scheme of the radiolabeled top strand fragment used in the DNase I footprinting assays in shown in the top diagram. DNA fragments assayed were isolated from plasmids pPK13116 [wild‐type P*torT*; lanes 1–4], pPK13248 [strengthened P*torT* containing an extended −10 element; lanes 5–8], and pPK13258 [strengthened P1*torS* with improved −10 element and disrupted P*torT* −35 element, corresponding to P*torT* ^(−36TTG‐34 to AAC/−10GC−9 to TT)^; lanes 9–12]. All fragments contained the P2*torS* ^(A‐11T)^ mutation. The presence of IscR or RNAP‐σ^70^ in the reaction is indicated by the + symbol. Orange and purple lines mark the regions of protection by RNAP‐σ^70^ in P*torT* and P1*torS*, respectively; the blue lines mark the region protected by IscR. Samples were electrophoresed with a Maxam‐Gilbert (A + G) ladder.
**Figure S7:** Western blot analysis to detect IscR protein levels in cells grown under aerobic (A) and anaerobic (B) growth conditions. Strains expressing chromosomally encoded *iscR* or the apoprotein derivative *iscR(C92A)* from P*tac* were grown in the presence of Tet to maintain a plasmid expressing LacI (pACYC*lacI* ^q^), and various concentrations of IPTG (μM). Below each band is the relative band intensity compared to that of IscR measured in a wild‐type strain, which was normalized to a value of 1.0; n.a. indicates not applicable.

## Data Availability

The data that supports the findings of this study are available in the Supporting Information of this article or upon request from the authors.
