# Supplementary material for: The Role of torS and torT Transcription in Driving a Bet‐Hedging Strategy for Trimethylamine N‐Oxide Reductase Expression in Escherichia coli
Source: Mol Microbiol. 2026 Aug 1;126(4):379–95. doi: 10.1111/mmi.70103 (PMC13644379; doi:10.1111/mmi.70103)
Supplement: Supplementary file 1 [file MMI-126-379-s001.docx]

**Supporting Figure 1.** β-galactosidase activity (Miller units) was measured in IscR^-^ (light gray bars) and IscR^+^ (dark gray bars) strains grown under aerobic conditions. Strains contained either a P*torS*-*lacZ* transcriptional fusion in which the sequence upstream of *torS* encompassing P1 and P2 replaced the native *lac* promoter, or a transcription fusion in which *lacZ* was inserted after the native *torS* coding sequence on the chromosome. The Miller units measured are indicated above each bar.

**Supporting Figure 2.** Identification of the *torT* transcription start site. The *torT* TSS was determined by primer extension using RNA prepared *in vitro* in which supercoiled plasmid DNA (pPK12347) harboring the sequence upstream of *torT* encompassing -140 to +40 bp relative to the predicted TorT start codon served as template. The products produced from DNA sequencing of pPK12347 are shown in lanes 1-4, and primer extension reactions with the same radiolabeled primer are shown in lane 5. For reference, partial sequence of P*torT* is shown, with the arrow marking the major TSS identified in this experiment (T). The gel image was digitally altered to remove a lane that was originally between lanes 4 and 5.

**Supporting Figure 3.** Promoter mutation analysis for P*torT* was carried out using *in vitro* transcription assays. Serving as DNA templates were supercoiled plasmids harboring the control RNA-1 promoter and either wild-type P*torT* (-120 to +60 bp relative to the *torT* TSS) or variants containing the indicated mutations relative to the *torT* TSS. All templates contained the P2*torS* ^(A-11T)^ mutation. Transcription products from each promoter are indicated. Templates included pPK13116 [wild-type], pPK13244 [disrupted P1*torS* -35 element corresponding to P1*torS*^(-35TAG-33 to AAC)^], pPK13245 [disrupted P*torT* -35 element], pPK13255 [disrupted -35 elements in both P*torT* and P1*torS*], pPK13246 [strengthened P*torT* with 17 bp spacing between -10 and -35 elements], and pPK13248 [strengthened P*torT* containing an extended -10 element]. The gel image was digitally altered to remove a lane that was originally between those labeled ∆G^-22^ and ^-15^AT^-14^ to TG.

**Supporting Figure 4.** Promoter mutation analysis was carried out by measuring β-galactosidase activity (Miller units) from variants of P*torT*-*lacZ* (A) and P1*torS*-*lacZ* (B) in IscR^-^ strains grown under aerobic conditions. Partial sequence of the 180 bp promoter fragment upstream of *lacZ* on the chromosome are shown (-60 to +19 bp relative to the P*torT* TSS; -36 to +43 bp relative to the P1*torS* TSS). With exception of fragment 1, P2*torS* was inactivated in all fragments [P2*torS*^(A-11T)^]. The orange and blue arrows mark the TSS of the promoter driving *lacZ* expression, mutations are boxed in red, and the IscR binding site is boxed in gray. The -10 and -35 RNAP-σ^70^ recognition elements are underlined for P1*torS* (single), P2*torS* (double), and P*torT* (dotted).

**Supporting Figure 5.** RNAP-σ^70^ provides little protection of P1*torS* from DNase I cleavage. The general scheme of the radiolabeled bottom strand fragment used in the footprinting assays is shown in the top diagram. DNA fragments assayed were isolated from plasmids pPK13116 [wild-type P*torT*; lanes 1 and 2], pPK13244 [disrupted P1*torS* -35 element corresponding to P1*torS*^(-35TAG-33 to AAC)^; lanes 3 and 4], pPK13248 [strengthened P*torT* containing an extended -10 element; lanes 5 and 6], pPK13245 [disrupted P*torT* -35 element, corresponding to P*torT*^(-36TTG-34 to AAC)^; lanes 7 and 8], pPK13258 [strengthened P1*torS* with improved -10 element and disrupted P*torT* -35 element, corresponding to P*torT*^(-36TTG-34 to AAC/-10GC-9 to TT)^; lanes 9 and 10], and pPK13255 [disrupted -35 elements in both promoters, corresponding to P*torT*^(-36TTG-34 to AAC/+16CTA+18 to GTT)^; lanes 11 and 12]. All fragments contained the P2*torS* ^(A-11T)^ mutation. The presence of RNAP-σ^70^ in the reaction is indicated by the + symbol. Orange and purple lines mark the regions of protection by RNAP-σ^70^ in P*torT* and P1*torS*, respectively. Position of DNase I enhancement is noted at position -25 relative to the P*torT* TSS. Samples were electrophoresed with a Maxam-Gilbert (A + G) ladder.

**Supporting Figure 6.** IscR binding to either P*torT* or P1*torS* prevents promoter recognition by RNAP-σ^70^. The general scheme of the radiolabeled top strand fragment used in the DNase I footprinting assays in shown in the top diagram. DNA fragments assayed were isolated from plasmids pPK13116 [wild-type P*torT*; lanes 1-4], pPK13248 [strengthened P*torT* containing an extended -10 element; lanes 5-8], and pPK13258 [strengthened P1*torS* with improved -10 element and disrupted P*torT* -35 element, corresponding to P*torT*^(-36TTG-34 to AAC/-10GC-9 to TT)^; lanes 9-12]. All fragments contained the P2*torS* ^(A-11T)^ mutation. The presence of IscR or RNAP-σ^70^ in the reaction is indicated by the + symbol. Orange and purple lines mark the regions of protection by RNAP-σ^70^ in P*torT* and P1*torS*, respectively; the blue lines mark the region protected by IscR. Samples were electrophoresed with a Maxam-Gilbert (A + G) ladder.

**Supporting Figure 7.** Western blot analysis to detect IscR protein levels in cells grown under aerobic **(A)** and anaerobic **(B)** growth conditions. Strains expressing chromosomally encoded *iscR* or the apoprotein derivative *iscR(C92A)* from P*tac* were grown in the presence of Tet to maintain a plasmid expressing LacI (pACYC*lacI*^q^), and various concentrations of IPTG (μM). Below each band is the relative band intensity compared to that of IscR measured in a wild-type strain, which was normalized to a value of 1.0; n.a. indicates not applicable.
